# Retraction Note: SLFN5 promotes reversible epithelial and mesenchymal transformation in ovarian cancer

**DOI:** 10.1186/s13048-024-01574-2

**Published:** 2024-12-11

**Authors:** Qiao Ping Xu, Kui Deng, Zhen Zhang, Hongkai Shang

**Affiliations:** 1 Department of Clinical Pharmacology, Key Laboratory of Clinical Cancer Pharmacology and Toxicology Research of Zhejiang Province, Affiliated Hangzhou, First People’s Hospital, Hangzhou, 310006 China; 2 Westlake Institute for Advanced Study, Zhejiang, Hangzhou, 310024 China; 3 Department of Oncology, Hangzhou Cancer Hospital, Zhejiang, Hangzhou, 310002 China; 4 Department of Gynecology, Affiliated Hangzhou First People’s Hospital, Zhejiang University School of Medicine, Zhejiang Province, Hangzhou, 310006 China

**Retraction Note: Journal of Ovarian Research (2023) 16:33**

10.1186/s13048-023-01103-7

The Editors-in-Chief have retracted this article after the authors were unable to provide appropriate documentary evidence of the ethics approval regulating this research. The Editors-in-Chief are no longer confident that this study complies with the journal’s ethics guidelines. None of the authors have responded to correspondence from the Publisher about this retraction.

